# The conserved protective cyclic AMP-phosphodiesterase function PDE4B is expressed in the adenoma and adjacent normal colonic epithelium of mammals and silenced in colorectal cancer

**DOI:** 10.1371/journal.pgen.1007611

**Published:** 2018-09-06

**Authors:** Jennifer K. Pleiman, Amy A. Irving, Zhishi Wang, Erik Toraason, Linda Clipson, William F. Dove, Dustin A. Deming, Michael A. Newton

**Affiliations:** 1 McArdle Laboratory for Cancer Research, Department of Oncology, University of Wisconsin–Madison, Madison, Wisconsin, United States of America; 2 Laboratory of Genetics, University of Wisconsin–Madison, Madison, Wisconsin, United States of America; 3 Molecular and Environmental Toxicology Center, University of Wisconsin–Madison, Madison, Wisconsin, United States of America; 4 Department of Statistics, University of Wisconsin–Madison, Madison, Wisconsin, United States of America; 5 Carbone Cancer Center, University of Wisconsin–Madison, Madison, Wisconsin, United States of America; 6 Division of Hematology and Medical Oncology, Department of Medicine, University of Wisconsin–Madison, Madison, Wisconsin, United States of America; 7 Department of Biostatistics and Medical Informatics, University of Wisconsin–Madison, Madison, Wisconsin, United States of America; Seattle Children’s Research Institute, UNITED STATES

## Abstract

Conservation over three mammalian genera—the mouse, rat, and human—has been found for a subset of the transcripts whose level differs between the adenoma and normal epithelium of the colon. *Pde4b* is one of the triply conserved transcripts whose level is enhanced both in the colonic adenoma and in the normal colonic epithelium, especially adjacent to adenomas. It encodes the phosphodiesterase PDE4B, specific for cAMP. Loss of PDE4B function in the *Apc*^*Min/+*^ mouse leads to a significant increase in the number of colonic adenomas. Similarly, *Pde4b*-deficient *Apc*^*Min/+*^ mice are hypersensitive to treatment by the inflammatory agent DSS, becoming moribund soon after treatment. These observations imply that the PDE4B function protects against *Apc*^*Min*^-induced adenomagenesis and inflammatory lethality. The paradoxical enhancement of the *Pde4b* transcript in the adenoma versus this inferred protective function of PDE4B can be rationalized by a feedback model in which PDE4B is first activated by early oncogenic stress involving cAMP and then, as reported for frank human colon cancer, inactivated by epigenetic silencing.

## Author summary

We have used the extensive genetic variation between genera within the mammalian order–mouse *versus* rat *versus* human–to discover genes whose broadly conserved transcripts differ in level in early colonic tumors compared to the normal colonic epithelium. Then, we developed a quantitative functional analysis using a targeted inactivating mutation in a genetically homogeneous strain, the congenic C57BL/6J *Apc*^*Min/+*^ mouse. This physiological genetic analysis of cancer involves parsing gene action into positive versus negative effects on the cancer of interest. Combining our studies with reports in the literature, we deduced that, by catabolizing cyclic AMP, the phosphodiesterase encoded by the gene of interest, *Pde4b*, protects against the early stages of colon cancer in the mouse. In advanced colon cancer in human patients, this gene is silenced, losing its inferred protective role. Together, this study illustrates the power of combining discovery by conservation among diverse mammalian genera with functional analysis within a single experimental species to understand a novel facet of human cancer.

## Introduction

The use of animal models is furthering our understanding and management of colon cancer as are genomic analyses of human colonic cancers. Animal models provide an under-appreciated advantage not found in the deep-sequencing studies of humans, though. By examining adenomas in mice and rats genetically predisposed to develop colon cancer, we can gain insights from the evolutionary divergence of these species that extend to human cancers. This study explores the strategy of using a pair of animal models for early human colon cancer as sources of information evolutionarily divergent from the human. We assess the power of the transcriptome in each model to predict the transcriptome of the corresponding neoplastic change in the human. We explore the possibility that the power of this test is maximized by using two models, the mouse and the rat, from distinct genera, *Mus* and *Rattus*, respectively. The *Apc*^*Min/+*^ mouse [1], is heterozygous for a nonsense mutation in codon 850 of the *Apc* “gatekeeper” gene [2]. On the C57BL/6J genetic background, the small intestine of the *Apc*^*Min/+*^ mouse develops 92 ± 33 adenomas in males and 103 ± 32 adenomas in females. By contrast, in the colon males develop only 2.9 ± 1.8 adenomas and females 1.6 ± 1.2 adenomas [3]. The *Apc*^*Pirc/+*^ rat is heterozygous for a nonsense mutation in codon 1137 of *Apc* [3]. On the F344/Tac genetic background, the small intestine of the *Apc*^*Pirc/+*^ rat develops only 17 ± 7 adenomas in males and 1.9 ± 1.6 adenomas in females. By contrast, in the colon it develops 20 ± 8.9 adenomas in males and 9.2 ± 6.0 adenomas in females [4]. Since the colon is the predominant site of intestinal neoplasia in the human, the transcriptomes of each genus were analyzed only from colonic adenomas.

This study aimed first to seek molecular signals of early colonic neoplasia that are conserved across genera, and then to ascertain which signals are also expressed in the apparently normal colonic epithelium, especially that adjacent to emergent tumors. Early studies of epithelial cancers by clinicians indicated changes occurred in normal epithelial tissue prior to tumorigenesis. The term Field Cancerization refers to “the constellation of locoregional changes triggered by long-term exposure of a field of tissue to a carcinogen, which is not necessarily recognized histologically; the remaining ‘field,’ despite adequate resection, is grossly normal but more susceptible to future insult” [5, 6]. Observations interpreted as reflecting field cancerization have been made in most epithelial cancers, including colon cancer [7–11]. Pre-neoplastic mutations in the underlying colonic mucosa could serve as a tumor-enhancing field effect [12].

Tissue inflammation can be considered as one possible source for field cancerization [13]. Inflammatory bowel diseases (IBD), such as Crohn’s disease and ulcerative colitis, strongly increase a person’s risk of developing colorectal cancer. The lifetime risk of developing colorectal in the United States is 5%, with the average age of diagnosis 70 years of age [14]. With an age of onset of IBD between 10 and 30 years of age, an affected person’s risk of developing colorectal cancer increases to 8% 20 years later and 18% 30 years later [15]. Thus, by affecting the entire colon, perhaps by inducing the formation of mutagenic reactive oxygen species, inflammation may cause field cancerization, strongly influenced by environmental exposure.

Thus, on one hand, the investigation of signals expressed in tumors that are also expressed in adjacent normal tissues can identify pro-tumorigenic causes of field cancerization. On the other hand, such signals can provide evidence for regionally expressed protective functions. Here too, an animal model whose genome can be manipulated provides the power to study *in vivo* the consequences of a particular genetic loss-of-function. This study investigates by mutational analysis in the mouse whether PDE4B, the product of one of the triply conserved molecular transcriptome signals detected in *Apc*^*Min/+*^ adenomas and in the adjacent normal colonic epithelium, acts positively or negatively on adenomagenesis.

PDE4B is specifically relevant to the issue of a sensitizing versus protective field effect in human colon cancer because it has been reported to be expressed in “non-neoplastic appearing colonic mucosa from patients with colorectal neoplasia” [16]. For the human, the emergent resources of molecular data for colorectal cancer in patients [17–19] enables an assessment of the mutational and expression status of the *PDE4B* gene in frank human colon cancer.

## Results

### Analysis by array hybridization of the levels of transcripts in colonic adenomas

**Fig 1:**
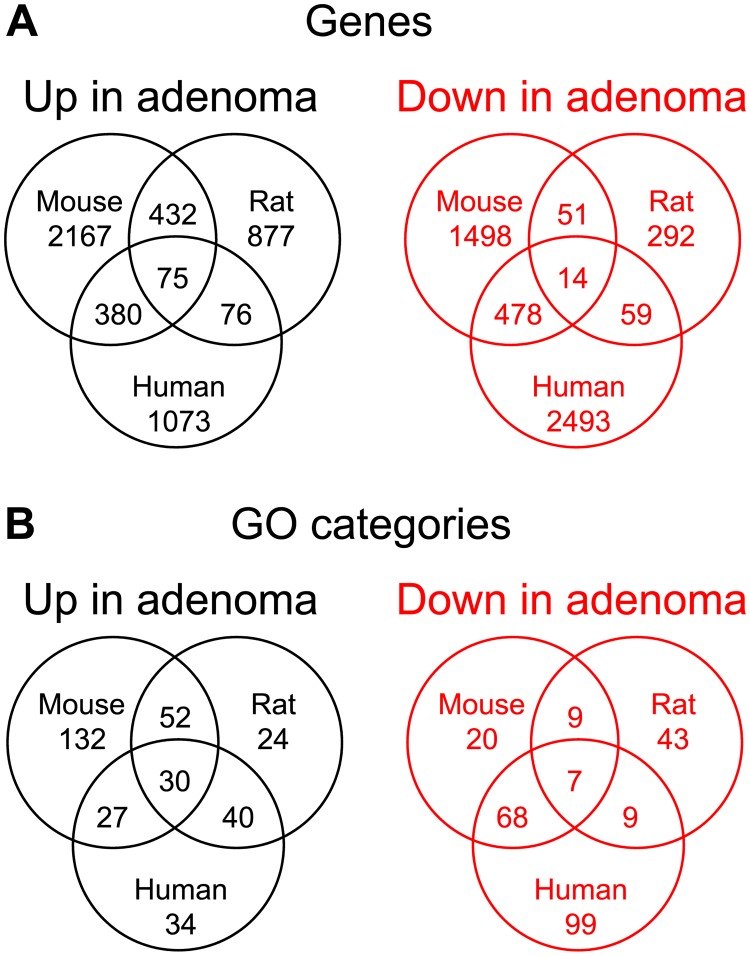
Venn diagram of genes whose transcript levels differ between colonic adenoma and normal epithelium for three genera. The sets of genes with differential transcript levels between colonic adenomas and normal colonic epithelium are summarized for the untreated *Apc* ^*Min/+*^ mouse, *Apc* ^*Pirc/+*^ rat, and human. Up in adenoma presents genes with increased levels in adenomas compared to the normal colonic epithelium. Down in adenoma presents genes with decreased levels in adenomas. The mouse and rat data were collected from experiments described in Methods and accessible in GEO datasets GSE107139 and GSE54036. Human data were retrieved from the published GEO dataset GDS2947 comparing adenomatous tumors with normal colonic tissue from 32 patients with spontaneous, non-familial colorectal cancer. For each genus, the comparison of transcript level was performed using a stringency of a change in transcript level by at least a factor of 2 with a false discovery rate of 0.05. The pairwise and three-way intersections for these sets were determined from this Venn diagram. The Venn diagram in Panel A displays the pairwise and three-way intersections of these gene lists between genera. For each differential gene list, the corresponding set of enriched GO categories was determined using a multi-set approach that accounts for category size variation and category overlaps. The Venn diagram in Panel B displays the pairwise and three-way intersections between genera of these lists of enriched GO categories. The higher agreement between genera at the GO level, suggested by these Venn diagrams, was tested by permutation analysis in Fig 2.

As described in Materials and Methods, RNA was isolated from colonic tumors and adjacent normal colonic epithelium of four *Apc*^*Min/+*^ mice and five *Apc*^*Pirc/+*^ rats. RNA populations were then analyzed by hybridization to Agilent microarrays. Human data were analyzed from published data comparing normal and adenoma tissue from 32 patients with spontaneous, non-familial colorectal cancer. For comparison purposes, transcripts were linked among genera using orthology information. Using analysis-of-variance methods for paired tumor-normal data we identified transcripts whose levels differ substantially between the colonic adenoma and its corresponding normal epithelium (Fig 1). In the colonic adenoma of the *Apc*^*Min/+*^ mouse, 3054 gene transcripts were enhanced and 2041diminished by at least a factor of 2, with a 5% false discovery rate. For the colonic adenoma of the *Apc*^*Pirc/+*^ rat, 1460 distinct transcripts were enhanced and 416 diminished by the same criteria. The difference between the mouse and rat in the numbers of transcripts with differences in level may be owing either to the noise characteristics of the expression data or to the incomplete annotation of the rat genome (rn5 Mar. 2012 RGSC Rnor_5.0) compared to the mouse genome (mm10 Dec. 2011 Genome Reference Consortium GRCm38). Though we did not pre-filter array data by removing low-expressing genes, the adenoma-associated transcripts showed substantially higher expression level than typical genes (S1 and S2 Figs) Finally, in the spontaneous human colonic adenoma 1604 annotated transcripts were enhanced and 3044 diminished (hg18 Mar. 2006 NCBI Build 36.1).

### Sharing between genera of transcripts with differences in level—By gene and by GO category

**Fig 2:**
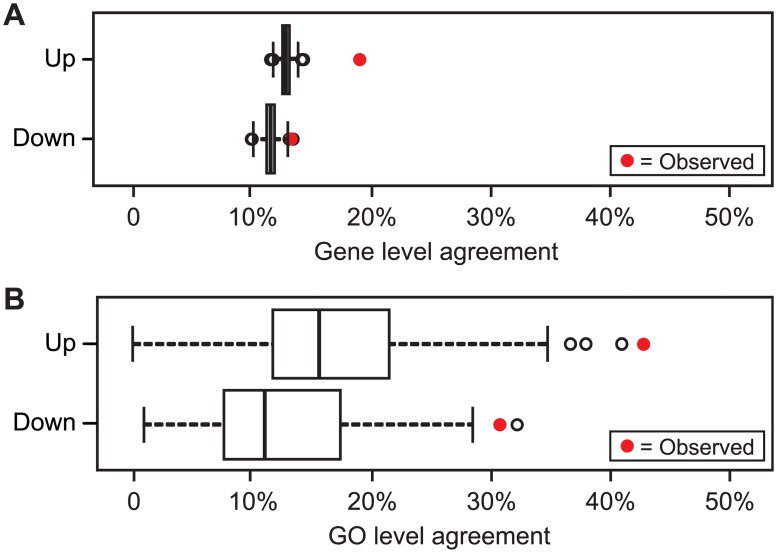
Permutation test of significance of agreement between differential transcriptome signals shared by all three genera. Each panel compares the observed mean pairwise overlap fraction (red) of the three species lists with the distribution of sharing rates for randomized data (boxplot, from 1000 gene-permutations). The extent of sharing among gene lists (Panel A) is lower than the GO level (19% and 13%, for up and down, respectively), but still higher than expected by chance. On a GO basis (Panel B), the observed intersection between genera for enhanced signals is 43% (p = 0.001) and for diminished signals 33% (p = 0.002). Though relatively few adenoma-associated transcripts are shared among all three genera, the agreement is statistically significant and is accentuated at the level of GO functional categories.

This three-genus study provides a unique window into conserved oncogenic processes. As we illustrate in this initial study, these conserved aspects of human adenoma formation can subsequently be analyzed further with targeted functional genetic studies in mice and/or rat models. To assess gene-level conservation, we determined the pairwise overlap between genera of individual genes whose transcripts show differences in level (Fig 1A). For example, on average over pairings of genera, the enhanced transcripts of one genus overlap with 19% of the enhanced transcripts of a second genus. For the transcripts that are diminished in adenomas, this mean overlap fraction is 13%. When considering the genome size for each of the three genera, this agreement in directionality of expression is significantly higher than would be expected in randomly generated lists of the same sizes (by Fisher’s Exact Test; p < 10^-10^ in mouse by human and mouse by rat; but p = 0.06 in rat by human comparisons). More directly, Fig 2A reports on a permutation test, as described in Materials and Methods, of the mean-overlap statistic, confirming that the 3-way agreement, while low, is significantly higher than expected by chance (p = 0.001, for enhanced in adenoma; p = 0.003, for diminished in adenoma).

It is well known that sources of variation at the gene level can erode measures of agreement between genome-wide profiles of *bona fide* linked biological processes. To assess the conservation of adenoma-associated processes between mouse, rat, and human, we then determined the pairwise sharing between genera at the level of Gene Ontology (GO) categories, using the approach pioneered by Hao [20]. Thus, we identified genus-specific lists of GO categories enriched for adenoma-associated genes, and made pairwise comparisons of these gene-category lists. In contrast to the gene-level analysis, the mean pairwise overlap calculated directly from the GO analysis (Fig 2B) far exceeded that calculated at the gene-level: 43% for the enhanced and 33% for the diminished categories.

**Fig 3:**
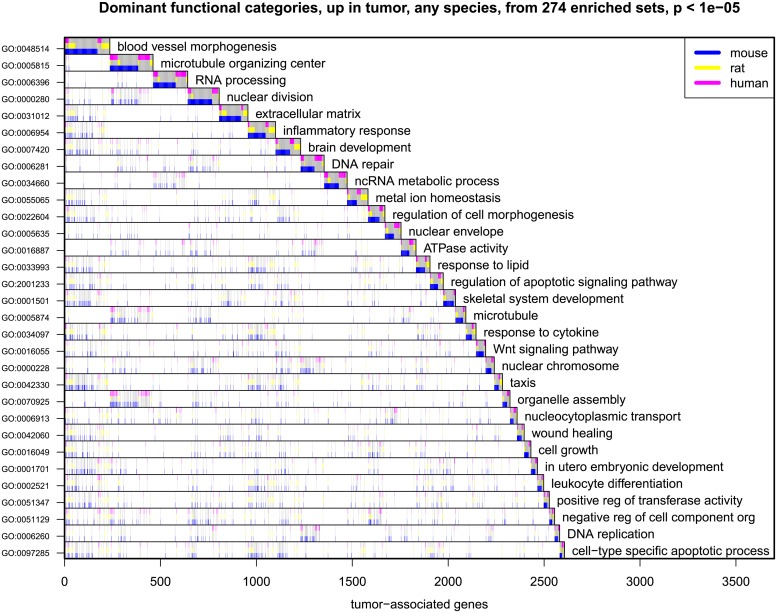
Dominant functional categories of the genes whose transcript levels are enhanced in adenomas of any of the three genera—Mouse, rat, and human. Dominant functional categories (rows) enriched in the large list of 5602 genes (columns) having increased transcript levels in tumors in any one of the three genera–mouse, rat, and human from among 19169 annotated genes. 274 populated GO sets showed enrichment in the union of gene lists at p < 10^−5^; a subset of these covering the union with low redundancy is shown in the Figure. Shaded area in each row counts the number of tumor-associated genes having that functional property; categories are ordered from the top by decreasing numbers of tumor-associated genes not contained in a prior category. Colors indicate the genus in which the transcript level is enhanced in adenomas.

**Fig 4:**
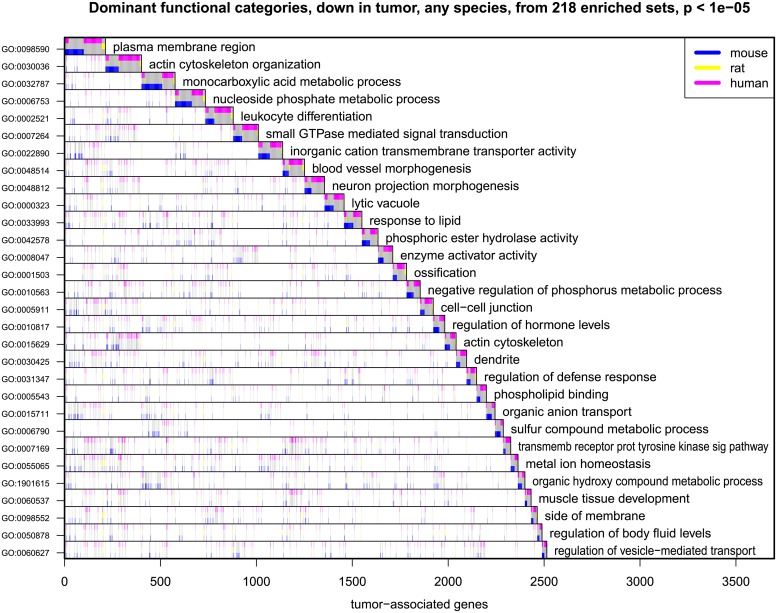
Dominant functional categories of the genes whose transcript levels are reduced in adenomas of any of the three genera—Mouse, rat, and human. Functional categories (rows) enriched in the large list of 5477 genes (columns) having decreased transcript levels in tumors in any one of the three genera–mouse, rat, and human from among 19169 annotated genes. 218 populated GO sets showed enrichment at p < 10^−5^; a subset of these covering the union with low redundancy is shown in the figure. The shaded area in each row counts the number of tumor-associated genes having that functional property; categories are ordered from the top by decreasing numbers of tumor-associated genes not contained in a prior category. Colors indicate the genus in which the transcript level is diminished in adenomas.

Transcripts represented in the union list of adenoma-association in at least one genus populate extended sets of GO categories (Figs 3 and 4). Speaking broadly, Fig 3 indicates that transcripts associated with angiogenesis, nuclear and cell division, RNA processing, extracellular matrix, and inflammation are enhanced in the adenoma. By contrast, Fig 4 indicates that transcripts diminished in the adenoma are associated with processes of differentiation such as monocarboxylic acid metabolism, the organization of the actin cytoskeleton and the plasma membrane.

**Fig 5:**
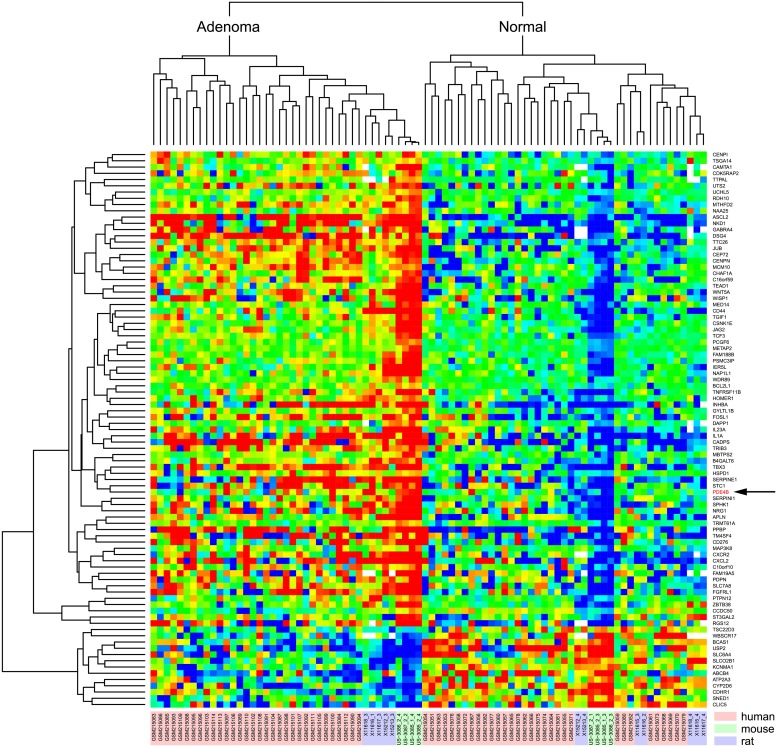
Heatmap summarizing the transcript levels from 89 genes (75 up; 14 down) that show altered expression in all three genera. Both the mouse and the rat models are genetically homogeneous, whereas the human is not. Thus, genetic heterogeneity can explain the dispersion of the human data. Color indicates deviation from gene- and genus-specific average expression (log2 scale). The arrow notes the position of the *Pde4b/PDE4B* transcript.

**Fig 6:**
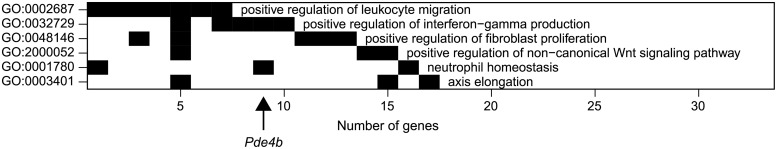
Dominant functional categories (GO terms) enriched in the 75 genes with increased transcript level in all three genera. Functional categories (rows) enriched in the75 genes (columns) having increased transcript levels in tumors in all three genera–mouse, rat, and human from among 19169 annotated genes. Shaded area in each row counts the number of tumor-associated genes having that functional property; categories are ordered from the top by decreasing numbers of tumor-associated genes not contained in a prior category.

We went on to focus on genes and their GO categories with differential levels in the adenoma shared across all three genera. The across-genus heat-map of Fig 5 summarizes the array data from the 89 genes that showed differences in level in a consistent direction in the colonic adenoma transcriptomes of all three genera– 75 enhanced and 14 diminished. Despite the genus differences, a clear across-genus adenoma-associated expression signature emerges. For the size of the 3-way intersection, the observed overlaps are significant at both the gene-level and the GO level. Here, the GO-level accentuates the conserved signal, focusing on shared fundamental biological processes in colonic adenomagenesis. We investigated whether the overlap statistics are sensitive to pre-filtering according to expression levels of the transcripts (S3 Fig) GO categories enriched in the three-way overlap are tabulated in S2 and S3 Tables, and summarized in Fig 6. For the triply conserved enhanced transcripts, we infer functions commonly assigned to the tumor microenvironment, including leukocyte migration and fibroblast proliferation. By contrast, the triply conserved GO categories for the diminished transcripts include functions involved in cellular response such as transmembrane transporter activity.

### Testing the functionality of *Pde4b* in the *Apc*^*Min/+*^ mouse

Finding a gene involved in colonic adenomagenesis in three distinct genera raises the hypothesis that the gene has an important function in this process. If so, by mutational analysis one can gain information relevant to function *in vivo*. Such molecular genetic tests of function by targeted mutagenesis are increasingly available in the mouse (through the Knockout Mouse Project: www.komp.org) and rat [21]. While it is not practical to analyze functionally all of the individual genes belonging to these significant triply shared GO categories, we have focused on *Pde4b*, one of the genes whose transcript is differentially enhanced in colonic adenomatous tissue of all three genera: 3.3-fold in the mouse, 2.7-fold in the rat, and 5.1-fold in the human. We have examined *Pde4b* in a mouse strain carrying its targeted knockout allele [22]. Phosphodiesterase 4b (PDE4B, encoded in the mouse by *Pde4b*) is a member of an eleven-member family of the cyclic nucleotide phosphodiesterases that enzymatically regulate the degradation of cAMP and cGMP. The *Pde4* gene family, specific for cAMP, is composed of four genes (A-D) transcribed into a number of splice variants [23, 24]. Little is known about PDE4B function in the intestine beyond basic expression profiles [23, 25].

The enhancement of *Pde4b* transcripts in colonic adenomas is consistent with, but does not prove, the hypothesis that the PDE4B function plays a pro-tumorigenic role in the colon. We asked: Does loss of PDE4B function reduce adenomagenesis in the *Apc*^*Min/+*^ mouse? To address this question, mice heterozygous for a global knockout allele of *Pde4b* [24] were crossed with *Apc*^*Min*/+^ animals. *Apc*^*Min*/+^ and *Apc*^+/+^ animals of *Pde4b*^+/+^, *Pde4b*^+/-^, and *Pde4b*^-/-^ genotypes were generated and the numbers of tumors in the small intestine and colon were scored. A significant effect of the *Pde4b* genotype was observed on the number of adenomas in the colon of the *Apc*^*Min/+*^ mouse (Table 1 and S4 Fig). The average numbers (± standard deviation) of colonic adenomas are 2.4 ± 2.2 for the *Pde4b*^*+/+*^, 3.3 ± 2.9 for *Pde4b* ^-/+^, and 4.3 ± 4.6 for the *Pde4b*^*-/-*^ genotype. By two-sided Wilcoxon rank sum test, the adenoma numbers in the heterozygote and homozygous mutant are significantly higher than in the wildtype (p = 0.004 and p = 0.0002, respectively), but do not differ significantly from each other (p = 0.06). Adenoma counts in the small intestine varied extensively for each *Pde4b* genotype; these differences were not significantly correlated with differences in the *Pde4b* genotype (S1 Table).

**Table 1:** Test of effect of the *Pde4b* genotype on the number of adenomas in the colon of *Apc* ^*Min/+*^ mice over a series of backcross-intercross generations. Gross tumor numbers were measured in the colons of *Apc*^*Min/+*^ mice from the series of backcross-intercross generations, carrying the *Pde4b*^*+/+*^, *Pde4b*^*+/-*^ and *Pde4b*^*-/-*^ genotypes. *Pde4b*^*+/+*^ animals (110) show the lowest average colon tumor count 2.4 +/- 2.2. *Pde4b*^*+/-*^ animals (192) show higher average colon tumor counts than *Pde4b*^*+/+*^ animals 3.3 ± 2.9. Finally *Pde4b*^*-/-*^ mice (66) show the highest average colon tumor number 4.3 ± 4.6. p = 0.0004 by the Kruskal-Wallis Test of association between tumor number and number of mutant alleles of *Pde4b*. We reject the null hypothesis that there is no difference in gross colonic tumor number as a function of *Pde4b* genotype.

Genotype	Colon tumor counts, mean ± SD (n of mice)		
	*Apc*^*Min/+*^ *Pde4b*^*+/+*^	*Apc*^*Min/+*^ *Pde4b*^*+/-*^	*Apc*^*Min/+*^ *Pde4b*^*-/-*^
F2	2.4 ± 1.5 (9)	3.1 ± 2.9 (18)	4.0 ± 2.2 (7)
N2F2	2.6 ± 2.4 (69)	3.1 ± 2.9 (124)	4.2 ± 5.2 (39)
N3F2	2.4 ± 2.1 (23)	4.2 ± 3.2 (33)	5.1 ± 4.3 (10)
N4F2	2.1 ± 2.3 (9)	2.9 ± 1.7 (17)	4.0 ± 3.7 (10)

In summary, this mutational analysis indicates that the *Pde4b* genotype significantly affects adenomagenesis in the colon of *Apc*^*Min/+*^ mice, even in heterozygotes for the knockout allele. The observed mutational enhancement of colonic adenomagenesis by a loss-of-function mutant allele is not consistent with a simple pro-tumorigenic role of wildtype PDE4B function. Instead, these observations imply that the wildtype *Pde4b* allele encodes a protective function.

### Enhanced levels of the *Pde4b* transcript in the normal colonic epithelium adjacent to colonic tumors—A protective field effect?

**Fig 7:**
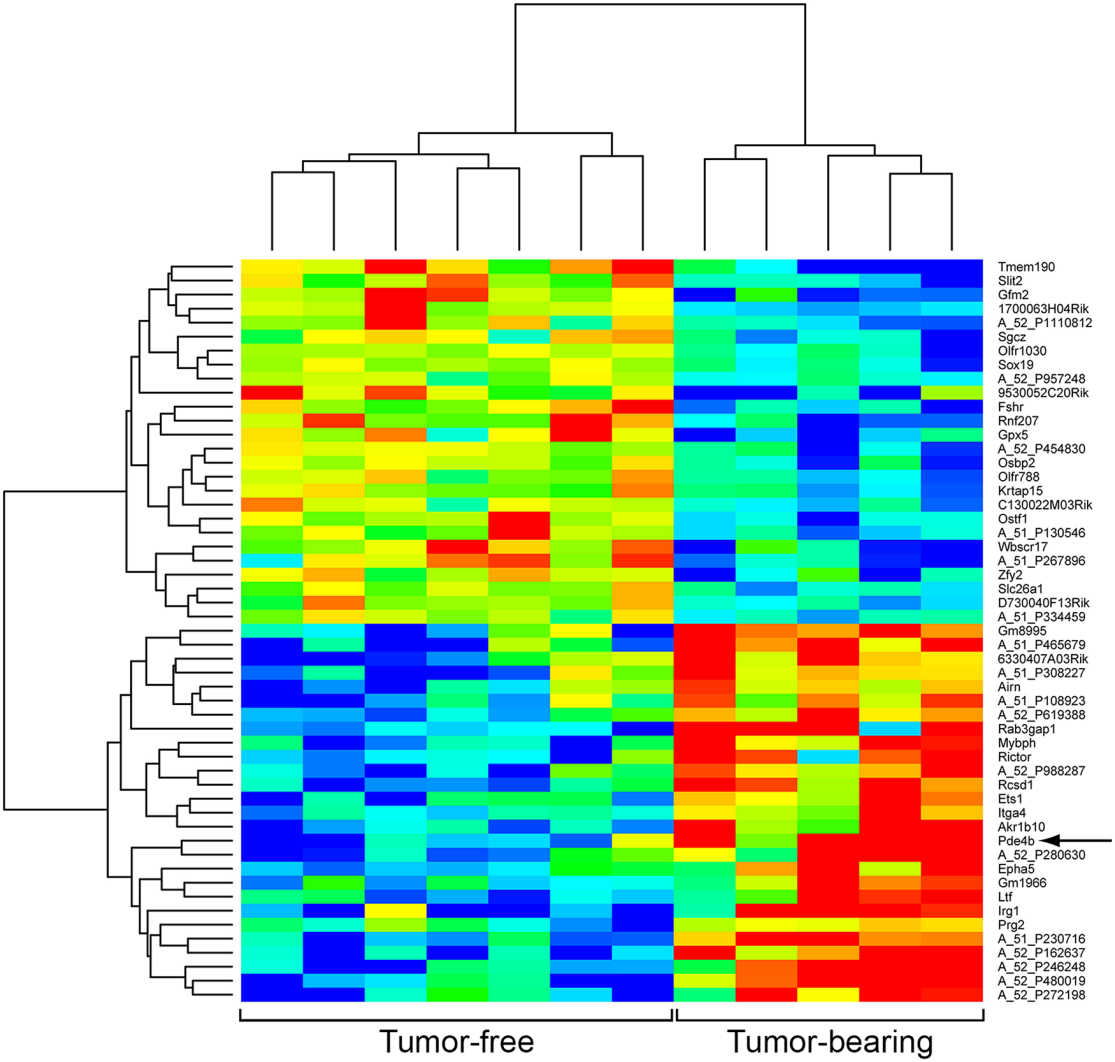
Heatmap summarizing the transcripts from the normal colonic epithelium of *Apc*^*Min/+*^ mice whose levels differ between tumor-bearing and tumor-free mice. Expression analysis was carried out for transcripts of the normal colonic epithelium of *Apc*^*Min/+*^ mice. The levels of these transcript populations were compared between mice bearing 1 or 2 colonic adenomas versus mice free of colonic adenomas. Analysis of differences between groups in hybridization signal was performed using EBarrays. The stringency cutoff was set at a difference of at least a factor of 2 fold and FDR of 20%. The *Pde4b* signal was confirmed by RT PCR analysis (Fig 9).

Because the average colonic tumor number in *Apc*^*Min/+*^ mice is small, a random subset of *Apc*^*Min/+*^ mice are free of colonic tumors, at least in the distal half of the colon. As described in Materials and Methods, they can be identified by endoscopy prior to necropsy. Accordingly, we compared the level of the *Pde4b* transcript in the normal colonic epithelium from tumor-free colons with that from tumor-bearing colons. By array analysis, we observed that the *Pde4b* transcript level is enhanced in the normal colonic epithelium adjacent to colonic tumors in *Apc*^*Min*/+^ mice, compared to that of the normal colonic epithelium of tumor-free *Apc*^*Min*/+^ mice (Fig 7).

### Confirmation of differential levels of the triply conserved *Pde4b* transcript in the colonic adenoma and adjacent normal colonic epithelium of *Apc* ^*Min/+*^ mice

**Fig 8:**
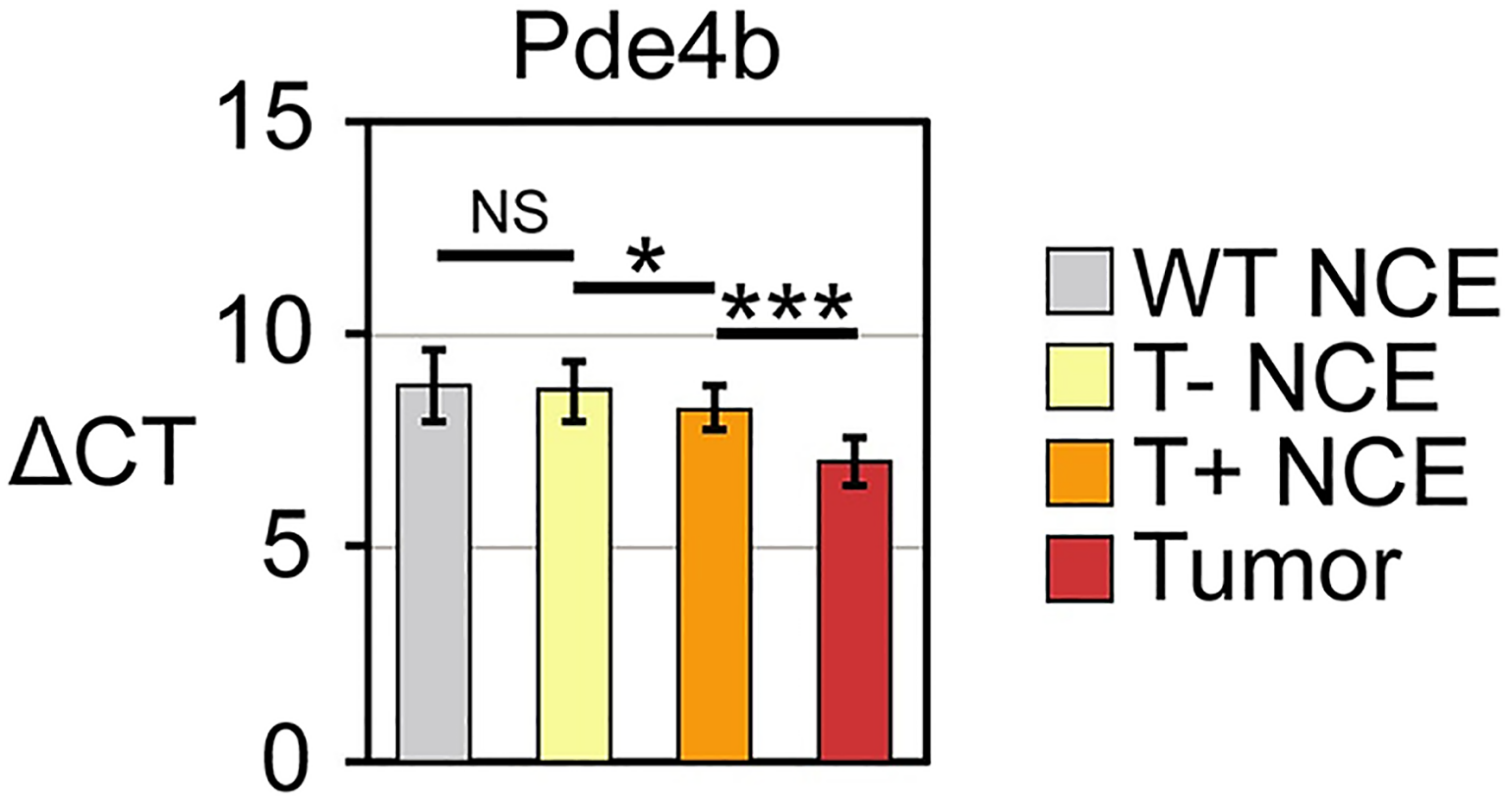
Real time PCR analysis of transcripts that differ in level between the *Apc*^*Min/+*^ adenoma and the normal colonic epithelium of tumor-bearing versus tumor-free *Apc*^*Min/+*^ mice. Real time PCR results for transcripts of the normal colonic epithelium (NCE) whose level differ between *Apc*^*Min/+*^ mice bearing colonic adenomas (T+) and *Apc*^*Min/+*^ mice free of colonic tumors (T-). The cycle number (ΔCT), normalized to GAPDH, is shown on the Y axis. The relevant significant pairwise comparisons are marked: * for p ≤ 0.05; ** for p ≤ 0.01; *** for p ≤ 0.001; NS for Not Significant. WT, wildtype.

The levels of the *Pde4b* transcript were quantified by real time PCR analysis, as described in Materials and Methods. Tumors express the *Pde4b* transcript at a level higher than in any other tissue examined (Fig 8, p = 0.00008). Compared to its level in the normal colonic epithelium of tumor-free colons of *Apc*^*Min/+*^ mice, this transcript is found at a 3.3-fold higher level in the adenoma and at a 2.1-fold higher level in the normal colonic epithelium of tumor-bearing colons (p = 0.048). The *Pde4b* transcript level in tumor-free *Apc*^*Min/+*^ mice shows no significant difference from that in the normal colonic epithelium of mice wildtype for *Apc* (p = 0.97).

### Effects of deficiency in *Pde4b* on inflammatory responses of *Apc*^*Min/+*^ mice

The functional importance of the triply conserved PDE4B function may extend beyond adenomagenesis. Inflammation is known to increase tumor multiplicity and tumor stage in colorectal cancer [26, 27]. Immune and inflammatory cells express high levels of PDE4B, thus reducing cAMP levels [28, 29]. Inhibitors specific for cAMP-specific phosphodiesterases have been used to treat other inflammatory conditions including chronic obstructive pulmonary disease (COPD) and asthma. This effect demonstrates the importance of cAMP-specific phosphodiesterases in enhancing the inflammatory process, providing a potential therapeutic target [30]. Further, intestinal tumorigenesis has a known inflammatory component, perhaps involving PDE4B function [24, 27, 31] On these bases, inactivating the PDE4B function in a colorectal cancer model of inflammation would be expected to attenuate or eliminate the enhancement of colonic adenomagenesis by inflammation.

To test this prediction, we generated *Apc*^*Min*/+^ and *Apc*^+/+^ animals bearing *Pde4b*^+/+^, *Pde4b*^+/-^, and *Pde4b*^-/-^ genotypes. Sets of mice with each *Pde4b* and *Apc* genotype were then divided into two groups, one treated with Dextran Sodium Sulfate (DSS), a model of inflammatory bowel disease (IBD), and the other left untreated. Colonic adenoma numbers and survival were then assessed.

**Fig 9:**
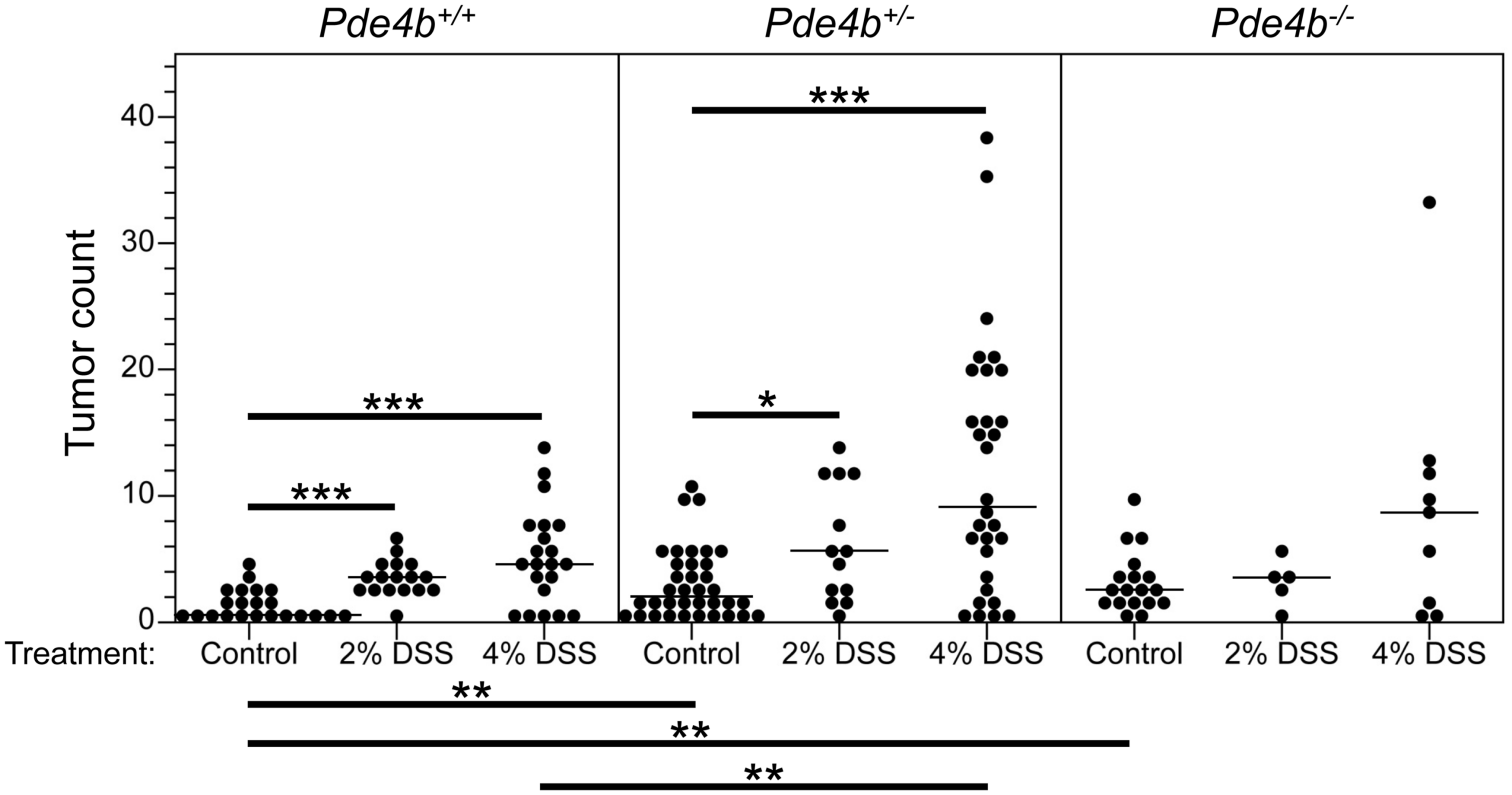
Test of the effect of *Pde4b* genotype on colonic adenoma numbers in *Apc* ^*Min/+*^ mice treated with DSS. *Apc* ^*Min/+*^ animals of *Pde4b*^*+/+*^, *Pde4b*^*+/-*^ and *Pde4b*^*-/-*^ genotypes treated with 2% and 4% DSS. Gross colonic tumor counts were measured in the colons. Two treated heterozygotes were sacrificed at 87 days; the treated homozygous mutants were sacrificed between 43 and 100 days. The other animals were all sacrificed at 100 days. The colonic tumor count of each animal is presented in this scatter plot. For the 2% DSS treatment, the numbers of mice and average colonic tumor multiplicity of each *Pde4b* genotype were: *Pde4b*^+/+^ animals (17) tumor count of 3.9 ± 1.4; *Pde4b*^+/-^ animals (13) tumor count of 6.5 ± 4.6; *Pde4b* ^-/-^ mice (5) tumor count of (3.6 ± 1.8). We cannot rule out the null hypothesis that there is no difference between gross colonic tumor number based on *Pde4b* genotype with 2% DSS treatment (p = 0.38 by the Kruskal-Wallis test). For animals treated with 4% DSS, 30 *Pde4b*^+/-^ animals show a higher average colon tumor count than 21 *Pde4b*^+/+^ animals (12.2 ± 9.9 versus 5.4 ± 3.9); p = 0.009 by the Wilcoxon rank sum test. Only 9 *Pde4b*^*-/-*^ animals entered the study; those colon tumor counts (9.7 ± 9.9) are not significantly different from counts for the other two genotypes. *, p ≤ 0.05; **, p ≤ 0.01; ***, p ≤ 0.001; two-sided Wilcoxon rank sum test. The median for each group is indicated by a thin horizontal line.

As shown in the left panel of Fig 9, *Apc*^*Min/+*^ mice that are wildtype for *Pde4b*, have significantly elevated colonic tumor counts after treatment with DSS. This enhancement is more extreme when *Apc*^*Min/+*^ mice are also heterozygous or homozygous for the knockout allele of *Pde4b* (Fig 9, central and right panels, respectively). This observation is inconsistent with the hypothesis that PDE4B function enhances the inflammatory pathway to colonic neoplasia. Instead, the wildtype phosphodiesterase acts to protect against DSS-associated colonic adenomagenesis—directly or indirectly. The involvement of the *Apc*^*Min/+*^ mutation in the lethal phenotype implies that *Pde4b* is a modifier of *Apc* in this protective function.

**Fig 10:**
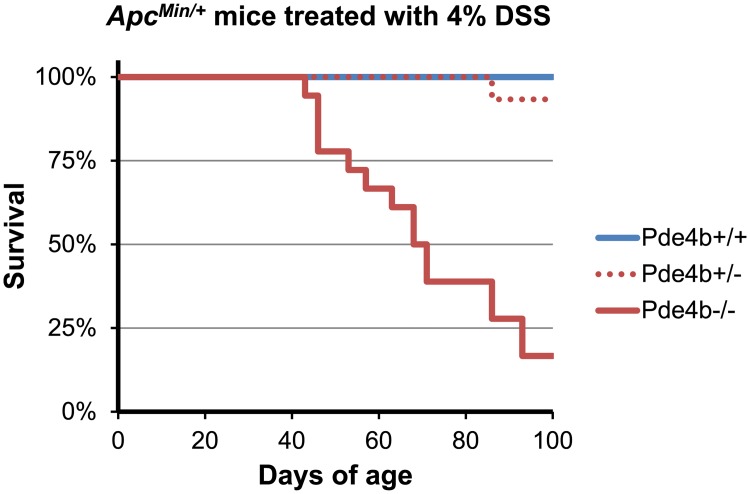
Kaplan-Meier survival curve of 4% DSS-treated *Apc* ^*Min/+*^ mice versus *Pde4b* genotype. The distribution of ages at which 4% DSS treated *Apc* ^*Min*/+^ mice of *Pde4b*^+/+^, *Pde4b*^+/-^ and *Pde4b*^+/-^ genotypes became moribund is presented as Kaplan-Meier survival curves. *Pde4b*^-/-^ mice demonstrate significantly accelerated morbidity compared to either *Pde4b*^+/-^ or *Pde4b*^+/+^ mice.

Beyond its effect on the number of DSS-induced colonic adenomas, we observed that the loss of PDE4B function has a severe effect on the survival of DSS-treated *Apc*^*Min/+*^ mutant mice. The imposition of the inflammatory pathway by treatment with 4% DSS, but not 2% DSS, compromises the survival of *Apc*^*Min/+*^ mice that are also mutated in the *Pde4b* gene (Table 2). This effect is specific to *Apc*^*Min/+*^ mice, and is most severe in homozygotes for mutated *Pde4b*. These effects are displayed graphically in the Kaplan-Meier plot of Fig 10. Once again, these observations support the hypothesis that, in *Apc*-mutant conditions, wildtype levels of PDE4B function are protective.

**Table 2:** Effect of the *Pde4b* genotype on the survival of *Apc* ^*Min/+*^ and *Apc* ^*+/+*^ mice treated with DSS. For each cohort of mice, the number surviving to 100 days of age is given in the numerator and the total number in the denominator.

Genotype	% survival to 100d (n/total)		
	4% DSS	2% DSS	No DSS
*Apc*^*Min/+*^ *Pde4b*^*+/-*^	93.3% (28/30)	100% (13/13)	100% (36/36)
*Apc*^*Min/+*^ *Pde4*^*-/-*^	16.7% (3/18)	100% (5/5)	100% (18/18)
*Apc*^*Min/+*^ *Pde4b*^*+/+*^	100% (21/21)	100% (17/17)	100% (23/23)
*Apc*^*+/+*^ *Pde4b*^*+/-*^	100% (3/3)	100% (3/3)	100% (6/6)
*Apc*^*+/+*^ *Pde4*^*-/-*^	100% (3/3)	100% (3/3)	100% (6/6)
*Apc*^*+/+*^ *Pde4b*^*+/+*^	100% (3/3)	100% (3/3)	100% (6/6)

## Discussion

Experimental animal models can provide a valuable tool toward understanding and managing human colon cancer. This study explored the strategy of using a pair of animal models–the mouse and the rat–together with the human, as evolutionarily divergent sources of information for the adenoma precursor to colon cancer.

### Functional annotation of the triply-conserved GO categories

Including *Pde4b*, we have found 89 triply conserved genes with differential levels of transcripts– 75 enhanced and 14 diminished in adenomas and have identified common functional categories associated with this conservation. Each of these differentially expressed, conserved genes can be analyzed as we have analyzed *Pde4b*. The differences in their transcript levels can result from either or both of two distinct causes: differences in transcript level per cell; and/or differences in the proportion of expressing cells in the adenoma *versus* the normal tissue [32]. Because the RNA samples were isolated from unfractionated tumor and epithelial tissues, changes in level can arise from the tumor lineage and/or the stromal microenvironment. Thus, we recognize the importance of assessing transcript levels in unfractionated adenoma tissue, to be able to identify cases of differential transcript level in either the tumor lineage or its stroma–“drivers”, “landscapers” [33], or other classes of “modifiers” [34, 35].

Conservation of transcriptome signals between evolutionarily divergent platforms may increase the likelihood of discovering functionally important signals. For example, the *Pde4b* gene has essential functions that protect against colonic adenomagenesis (Table 1) and DSS-associated adenomagenesis (Fig 9) and lethality (Fig 10 and Table 2). Our findings lead us to this hypothesis: molecular signals conserved at a particular neoplastic stage in multiple distinct animal models as well as in humans are more likely to identify fundamental functions that are involved in that specific stage in colonic neoplasia.

### PDE4B in adenomas and adjacent normal colonic epithelium

Changes in the levels or activity of PDEs have been implicated in diverse roles in multiple diseases, including cancer [36, 37]. cAMP is a critical mediator of multiple signaling events related to cell growth, differentiation, metabolism, and adhesion, among others. Its role in cancer is context dependent, as the cellular effect of a cAMP signal depends on the cell type and subcellular localization of the signal [36, 38]. In the select cases in which cAMP has been shown to be oncogenic [39], it may act directly on protein kinase A (PKA) or indirectly on its downstream effectors, cAMP-responsive element binding protein and Rap Guanine Nucleotide Exchange Factor 3 (RAPGEF3) [40].

**Fig 11:**
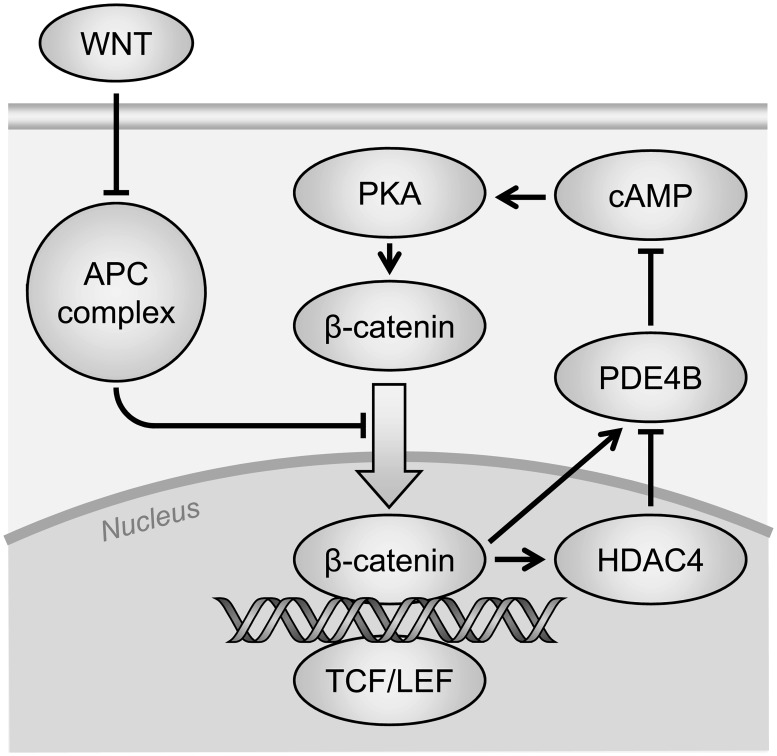
Feedback model for the interaction of Wnt and cyclic AMP signaling. The APC complex mediates the destruction of β-catenin. Its activity in turn is neutralized by WNT signaling allowing for β-catenin accumulation and nuclear translocation. This double-negative chain makes WNT a positive-regulator of β-catenin activity. When APC is truncated, increased β-catenin-mediated transcription ensues, causing tumorigenesis in the colon. Promoter-binding assays [51] are interpreted to show that nuclear β-catenin activates the transcription of *PDE4B*, whose hydrolytic activity inactivates cyclic AMP (cAMP). Since cAMP is a positive effector of protein kinase A, which in turn activates β-catenin, the hydrolysis of cAMP blunts the activation of β-catenin. The observed silencing of *PDE4B* in advanced colonic cancer, which would be predicted to enhance β-catenin activity, may involve the repressive histone deacetylase HDAC4, that is also β-catenin target gene.

The quantitative analysis of *Pde4b* transcripts by realtime PCR (Fig 8) showed levels in the *Apc*^*Min/+*^ adenoma enhanced by 3.3-fold over those in the normal colonic epithelium of wildtype and tumor-free *Apc*^*Min/+*^ mice. Contrary to the hypothesis that PDE4B function is pro-tumorigenic, we observed that *Apc*^*Min/+*^ animals carrying an inactivating mutation in *Pde4b* developed 1.4-fold more colonic adenomas in the mutant heterozygote and 1.8-fold more in the homozygote (Table 1). PDE4B functions directly or indirectly to inhibit colonic adenomagenesis in the *Apc*^*Min/+*^ mouse. This apparent paradox can be explained by a pro-tumorigenic effect mediated by cAMP, for example through activation of PKA as outlined above. In this scenario, loss of function of PDE4B would result in increased levels of cAMP, which in turn would activate PKA. Interestingly, PKA function has been shown to enhance the transcriptional activity of β-catenin by phosphorylating β-catenin on Ser675 [40, 41]. Therefore, reduction of both APC and PDE4B functions would jointly activate β-catenin and increase adenomagenesis in the *Apc*^*Min/+*^ mouse. This hypothesized negative feedback circuit is diagrammed in Fig 11.

We have found that *Pde4b* transcript levels are not only enhanced 3.2-fold in colonic adenomas, but also 2.1-fold in the normal colonic epithelium adjacent to tumors (Fig 8 and S5 Fig). Consistent with these observations, Mahmood and colleagues have observed by immunohistochemistry that PDE4B protein levels are enhanced in “non-neoplastic appearing colonic mucosa from patients with colonic neoplasia” [16]. These authors suggested that PDE4B is overexpressed as a malfunctioning protein in normal tissue. Instead, as suggested above, wildtype PDE4B functions as a negative regulator of colonic adenomagenesis. Its regional action formally fits one feature of the model of Meinhardt [42] that posits long-range negative coupled with short-range positive action in pattern formation. One is struck by the formal analogy with the long-range negative action of the metabolically stable angiostatin on tumor angiogenesis [43].

### Protection by PDE4B function against DSS-induced lethality in *Apc*-mutant mice

The compromised survival of DSS-treated *Apc*^*Min/+*^ mice carrying a knockout mutation in *Pde4b*, either as heterozygotes or more severely, as homozygous mutants (Fig 10 and Table 2) is striking. This effect on survival also requires the presence of the heterozygous *Min* nonsense mutation in the *Apc* gene, an example of “synthetic lethality” [44]. What model might explain this DSS-induced lethality? We reason that a function affecting survival at an early adult stage does not involve stochastic tumorigenesis, but instead is constitutional. The *Apc* gene is known to be expressed in most mammalian tissues. The interaction of DSS treatment with the *Apc*^*Min/+*^ heterozygous and *Pde4b* mutant conditions would rely on normal APC and PDE4B functions in survival of DSS treatment. Because the *Apc* gene is expressed broadly, any explanation of this DSS-dependent lethality would require comprehensive, timed necropsies, an analysis beyond this study.

### Effects of loss-of-function *Pde4b* alleles—The case for haploinsufficiency

The test of the function of PED4B in adenomagenesis showed strong enhancement in both the heterozygous and homozygous mutant *Pde4b* genotypes (Table 1), consistent with a protective role for PDE4B function. Under this interpretation, it seems paradoxical that the heterozygote, carrying one copy of the protective allele, is not significantly resistant. The sensitivity of the *Pde4b*^*+/-*^ heterozygote implies that PDE4B must function at a level higher than the 50% level expected for the heterozygote–“haploinsufficiency.” Indeed, the *Apc* gene also demonstrates haploinsufficiency for intestinal adenomagenesis [45] and other processes in mice [46–48]. To incorporate haploinsufficiency, the canonical Tumor Suppressor Model has been amended to a “one-hit model” by Berger, Knudson and Pandolfi [49].

### Removal of the functions of negative regulators over the stages of neoplasia

We have deduced that PDE4B function inhibits adenomagenesis, directly or indirectly. It then seems paradoxical that the levels of the *Pde4b* transcript are enhanced in colonic adenomas [50]. A possible resolution to this paradox comes from the observation that *Pde4b* is a target gene of β-catenin [51]. Thus, it is plausible that the increase of *Pde4b* transcript in the *Apc*^*Min/+*^ adenomas and surrounding mucosa is secondary to active WNT signaling in the setting of loss of APC. Then, through the destruction of cyclic AMP, PDE4B would act as a negative regulator of PKA-dependent colonic adenomagenesis (Fig 11).

We note that the normal, non-mutated form of another negative regulator, TP53, is also enhanced in early tumors [52, 53]. In frank cancers, then, the *p53* gene is frequently mutated. These observations gave support to the classical tumor suppressor model. At what stage in tumorigenesis does the negative regulation by the normal TP53 function act? For intestinal neoplasia in the *Apc*^*Min/+*^ mouse, it has been reported that *p53*-deficiency enhances adenomagenesis only 1.5-fold, leading to a small proportion of more advanced adenomas [54]. Thus, the protective function of TP53 may come into play at a stage later than that invoked in these studies of the PDE4B function. Does the overexpression in early tumors of the non-mutated normal forms of TP53 and PDE4B reflect a feedback response by normal tissues to uncontrolled growth–“oncogenic stress” [55, 56]? Two reports by Tomlinson and colleagues are relevant–TP53 protein levels are elevated only late in the progression of colon cancer [57] and the mutations in *p53* that are found in the adenomas of familial adenomatous polyposis patients are a special subset of those found in frank colon cancer [58].

Will frank human cancers show evidence for mutations in the *PDE4B* gene? In interrogation of the Dana Farber Cancer Institute (DFCI) dataset, *PDE4B* mutations are reported in only 9 of 619 colorectal cancers (1.5%) [17, 18, 59]. Of these mutant alleles, only one creates a known truncation, and none has been classified as a putative “driver” [cf. 60]. Many of these mutant alleles of *PDE4B* occur in colonic cancers that also carry truncations of the canonical gatekeeper gene *APC* [59]. A significant minority also carry putative driver mutations in *TP53* [59]. Interestingly, 40% of those cancers with mutant *PDE4B* also possess mutations in the *BRAF* gene, in contrast to an incidence of only 8–10% *BRAF*-mutant carriers in the entire colorectal cancer population.

Although rarely mutated in human colorectal cancers, the level of PDE4B protein is reduced in frank colon cancer—possibly through an epigenetic mechanism. Specifically, the Human Protein Atlas reports that 8 out of 12 human colorectal cancers demonstrate reduced intensity of immunostaining for PDE4B antigen in the epithelial component of these cancers, compared to samples from the normal colon and rectum [19]. These mutational and silencing observations in patients are consistent with PDE4B serving a protective function in the major APC-dependent pathway to frank colon cancer. In contrast to TP53, however, the function of PDE4B is commonly lost in colorectal cancer not by inactivating mutations but by epigenetic silencing. Consistent with this hypothesis, Bottomly and colleagues have reported that β-catenin strongly binds to the promoter region of the histone deacetylase HDAC4, a component of repressive chromatin [51]. An emergent challenge is to discern whether the inferred silencing process is stochastic or instead is developmentally programmed within the somatic lineage that leads to frank colorectal cancer [61]. A silencing process can be monoallelic or biallelic [61]. The observation that PDE4B function is haploinsufficient indicates that, though silencing may only be partial, it can be effective for PDE4B.

Fig 11 summarizes the interactions suggested in this Discussion. Here, the proposed negative regulatory role of HDAC4 would invoke several intriguing unknowns for further investigation: the developmental pathway or stochastic process that would uncover the silencing event and the specificity factors that would direct the repressive HDAC4 to the *Pde4b* gene

The observation that *Pde4b* affects the colonic adenoma phenotype of *Apc*^*Min/+*^ animals leads to its formal designation as a *Modifier of Min* (*Mom)* gene–one whose mutant phenotype depends also on the *Apc*^*Min/+*^ genotype. Most studies that define *Mom* loci involve measurements of tumor numbers in both the small intestine and colon. This study and that of the gender effect [3] show that these two regions of the intestinal tract differ in their response to genetic modifiers. How the understanding of the complexities of colon cancer being unraveled by mutational [62] and phenotypic [63] analysis can be conceptually simplified into metaphors including driver, landscaper and growth rate regulator [33, 64] is unclear. It seems possible that an indefinitely large number of functions can impact colonic neoplasia; such an “Infinite Tree” can be explored by the expansion of modifier genetics [62] and conservation of molecular signals across multiple genera. “Personalized Medicine” would benefit from such an expansion of functional targets.

In summary, we have combined mutational analysis in the *Apc*^*Min/+*^ mouse with published studies of frank colon cancer in patients to deduce that *Pde4b* has two strong biological functions. It negatively regulates colonic adenomagenesis in *Apc*^*Min/+*^ mice. In patients, PDE4B is most commonly inactivated by an epigenetic process. PDE4B protects against the lethality caused by treatment of young *Apc*^*Min/+*^ mice with the inflammatory agent DSS.

Finding evidence for important functions of PDE4B in tumorigenesis and survival supports applying the strategy of combining molecular analyses of human tumors with molecular and functional analyses of a pair of animal models from distinct genera to discover conserved functions that are important to understand and manage human cancer.

## Materials and methods

### Ethics statement

Mice and rats were maintained under a protocol (M02049-0-11-11 and M00268-0-07-13) approved by the Animal Care and Use Committee of the University of Wisconsin School of Medicine and Public Health, in a facility in the McArdle Laboratory approved by the American Association of Laboratory Animal Care. All experiments were carried out in accordance to the Guide for the Care and Use of Laboratory Animals from the National Research Council of the National Academies.

### Animals

Animals were housed in standard caging with free access to food (5020 chow, Purina, St. Louis, MO) and acidified water. A 12:12 hour light:dark cycle was maintained throughout the experiments. C57BL/6J *Apc*^*Min*/+^ mice (developed in the laboratory of WFD and commercially available through the Jackson Laboratory, Bar Harbor, ME) were maintained as a closed colony, C57BL/6JD [62] by breeding *Apc*^+/+^ females to *Apc*^*Min*/+^ males and monitoring the canonical high tumor number [1] (University of Missouri Stock 043849-MU). F_1_ generation (C57BL/6JD x BTBR)F_1_-Min mice were generated by breeding female C57BL/6JD *Apc*^*Min/+*^ to male BTBR mice. F_1_ generation (ACIxF344)-Pirc rats were generated by breeding female *ACI* Apc^+/+^ rats (Harlan, Indianapolis, IN) to male F344/Tac coisogenic *Apc*^*Pirc/+*^ rats (developed in the laboratory of WFD and commercially available through Taconic, Hudson, NY). The *Min* and *Pirc* alleles were genotyped as previously described [65, 66].

C57BL/6 *Pde4b*^*-/-*^ mice were generously contributed by the laboratory of Marco Conti (University of California San Francisco). Pde4B knockout mice were generated as described [67]. F_1_ mice were generated by breeding C57BL/6JD *Apc*^*Min*/+^ *Pde4b*^+/+^ females to C57BL/6 *Apc*^+/+^ *Pde4B*-/- males to generate C57BL/6JD *Apc*^*Min*/+^ *Pde4b*^+/-^ and C57BL/6 Apc^+/+^ *Pde4b*^+/-^ mice. F_2_ mice were generated by intercrossing F1 C57BL/6JD *Apc*^*Min*/+^ *Pde4b*^+/-^ and C57BL/6 *Apc*^+/+^ *Pde4b*^+/-^ mice. N_2_-N_5_ mice were generated by backcrossing C57BL/6 *Apc*^+/+^ *Pde4b*^+/-^ mice with C57BL/6JD *Apc*^*Min*/+^ *Pde4b*^+/+^ mice. Comparisons were made between mice of contrasting genotype from the same intercross generation.

### Dextran sodium sulfate treatment

At 35 days of age, sets of male and female F_2_ C57BL/6 *Apc*^*Min*/+^ animals of *Pde4b*^+/+^, *Pde4b*^+/-^, and *Pde4b*^-/-^ genotypes were divided into litter-matched or age-matched groups, with four animals in each cage. Dextran sodium sulfate (500kDa) was purchased from Fisher Scientific (Pittsburgh, PA) and mixed with standard acidified drinking water to 2% or 4% (wt/vol). The DSS-supplemented drinking water was administered to the treatment group between 35 and 39 days of age for four days, followed by a 17-day recovery period, after which they were given DSS for another four days between 56 and 60 days of age. Mice were sacrificed at 100 days of age or when moribund.

### Endoscopy

Beginning at 35–40 days of age, endoscopy was performed weekly until sacrifice, except during DSS treatment. This experimental approach allowed the selection of the subsets of tumor-positive and tumor-negative *Apc*^*Min/+*^ mice. For endoscopy, animals were anesthetized with 3% isoflurane and placed on a sterile surgical field, ventral side down. The colon was flushed with 1% saline to remove fecal material and provide lubrication. A Hopkins Optik 0° 10cm endoscope (1232AA, Karl Storz, Tuttlingen, Germany) contained within a sheath (61029D, Karl Storz) was inserted into the colon, allowing the distal half of the colon to be visualized. Still and video images were captured at each visit using a Xenon Nova 175 light source (20131520, Karl Storz) with an Image 1 hub (2220020, Karl Storz) and viewed using AidaVet software (69204020, Karl Storz). The final endoscopic visit was completed at least 24 hours prior to sacrifice.

### Dissection and terminal tumor counts

At sacrifice, the small intestine (divided into four equal sections) and colon were removed, opened longitudinally, laid flat, and washed with PBS. The four sections of small intestine and the colon were then fixed with 10% formalin for 48 hours and transferred to 70% ethanol for long-term storage. Following fixation, each section of intestine was viewed under 10X magnification on a dissecting microscope. Tumor counts were obtained for each of the four sections of small intestine and for the entire colon.

All analyses for differences in tumor multiplicities were done using Mstat software (https://mcardle.wisc.edu/mstat/index.html). Since the tumor distribution is known to be non-normal, non-parametric tests were used. A Kruskal-Wallis test was used for differences among tumor multiplicities over more than two sample groups. A two-sided Wilcoxon rank sum test was used to test for differences between two sample groups. For each test, a p-value ≤ 0.05 was considered significant.

### Microarray sample collection and preparation

Both the mouse and rat microarray studies utilized only male animals to eliminate potential hormone variation of the estrus cycle in female animals. A 12:12 hour light:dark cycle was maintained throughout the experiments and all tumors were harvested within a four-hour window in the afternoon to minimize any circadian cycle variation in transcript level. Only untreated mice and rats were included in this study; no chemical or biological mutagens were used. Thus the *Apc*^*Min*/+^ and *Apc*^*Pirc/+*^ mutations, respectively, were the sole controlled causes of adenoma development.

Untreated *Apc*^*Min/+*^ mice at 80 days of age and untreated *Apc*^*Pirc/+*^ rats at 97 days of age were sacrificed for RNA collection from adenoma and normal colonic tissue. Tumor samples were collected immediately upon dissection as follows: a cut was made part way down the middle of the tumor perpendicular to the surface of the colon. Then a second cut was made parallel to the surface of the colon, resulting in a segment representing approximately one quarter of the adenoma. Normal colonic epithelial tissue was collected by scraping the colonic lumen with a scalpel blade, at least 3mm from any visible tumor.

Each tissue sample was homogenized in a tube containing RLTplus buffer (Qiagen, Hilden, Germany) and frozen at -80°C. RNA was isolated from each sample using the Allprep DNA/RNA Mini Kit (Qiagen), following the manufacturer’s protocol. DNA contamination of RNA samples was removed by on-the-column DNase treatment following the manufacturer’s protocol. RNA quality was determined using an Agilent 2100 BioAnalyzer (Agilent Technologies, Inc. Santa Clara, CA, USA).

### Microarray design

Microarray experiments follow the nomenclature, descriptions, and data sharing protocols recommended by the MIAME Guidelines [68]. For each normal colonic epithelium and adenoma sample, total RNA (100 ng) was labeled with Cy3 dye using a Low Input Quick Amp kit (Agilent Technologies) according to the manufacturer’s instructions. Mouse samples were hybridized to Agilent 8x60K Whole Genome microarrays. Rat samples were hybridized to Agilent 4x44K Whole Genome microarrays. Following incubation, arrays were scanned on an Agilent High-resolution Microarray Scanner at 3μm resolution with a 20-bit data format. Files were extracted using Agilent Feature Extraction version 10.7.

### Microarray data summary

Human data were analyzed from the published GEO dataset GDS2947 comparing normal and adenoma tissue from 32 patients with spontaneous, non-familial colorectal cancer. Mouse data were acquired as documented in GEO Series accession number GSE107139: paired normal and tumor tissue was harvested from each of 4 Min mice. Rat data were acquired as described in GEO Series accession number GSE54036: paired normal and tumor tissue was harvested from each of 5 rats plus an additional normal sample from 2 of those rats.

### Statistical methods, within genus comparisons

Mouse and rat data from Agilent extraction files and human data from Affymetrix extraction files were analyzed using Genome Suite software (Partek). Samples from each genus were analyzed separately, using the same mode of analysis for each genus, to generate three lists of transcripts with differential levels. 2-Way ANOVA was used to assess differential levels, with animal ID and sample type (adenoma or normal colonic epithelium) as variables. Animal ID was included as a variable to permit a paired analysis of tumor and normal samples. Gene lists were generated from transcript levels that differed by at least a factor of 2 (up or down) between normal epithelium and adenoma, with a false discovery rate of equal to or less than 0.05. For comparison of differences of transcript levels of tumors and the normal colonic epithelium of tumor-bearing and tumor-free animals we used the LNNMV model in Bioconductor package EBarrays [69].

### Statistical methods, between genus comparisons

Orthology information relating mouse, human, and rat genes was obtained from the Mouse Genome Informatics Web (http://www.informatics.jax.org), retrieved April 2013 [70]. Within-genus differential expression results were aligned according to Entrez gene ID using mouse-rat and mouse-human orthology. To allow an equivalent comparison involving all genera, Entrez IDs orthology selected only the probes that were represented in all three genera.

Genus-aligned data were compared pairwise and over three genera by tabulating genes that were consistently up or down (adenoma versus normal epithelium) among genera. Pairwise overlap was measured with the mean-overlap fraction, which is the average proportion of one gene list that is contained in another, averaged over the different source lists [20]. Fisher’s exact test was also used as a baseline approach to assess whether the overlap between two lists was higher than expected from randomized gene lists of the same sizes. We also assessed 3-way overlap using permutations that shuffled gene-list content independently in each genus.

Gene-set analysis was used for multiple purposes: (1) to identify functional categories that were enriched in genus-specific gene lists; (2) to identify functional categories enriched in all-genera gene lists; and (3) to describe the among-genera agreement at the functional level. In all cases Gene Ontology (GO) classes mapping to the linking mouse Entrez-gene IDs were used from Bioconductor package org.Mm.eg.db (June 2015). Enrichment computations used both *allez* [71] and model-based multi-set computations [72]. Multi-genera agreement [73] was computed in a gene-permutation analysis using the technique described by Hao et al. [20]. Briefly, enriched GO terms (via model-based computation) were identified in each species, and the GO-term lists were compared for their mean-overlap fractions. For a comparison, the mean overlap fraction was also recorded on gene lists. These statistics were calibrated by gene-shuffling in which gene-set content was shuffled but the gene-list sizes and gene-level agreements were fixed among genera. An increase in overlap fraction among genera at the functional GO level, compared to the gene level, would signify pathway or functional agreement among genera in the level of transcripts associated with adenomagenesis. This agreement is masked by gene-level noise.

Dominant functional categories (Figs 3, 4 and 6,) were derived from enriched categories and plotted using the same technique as in Hao et al. [20] and Barger et al. [74]

### RT-PCR

Transcriptome candidates were verified by real time PCR using experiments following the nomenclature and description recommended by the MIQE Guidelines [75]. cDNA was generated from isolated RNA using the Superscript III Reverse Transcriptase Kit (Thermo Fischer Scientific, Waltham, MA). The hydrolysis probe labeled with FAM dye for *Pde4b* was purchased from Applied Biosystems. A GAPDH probe labeled with VIC dye (Applied Biosystems, Foster City, CA) was used as a reference gene. Normal colonic epithelial samples of both tumor-bearing and tumor-free *Apc*^*Min/+*^ animals were analyzed for all array samples. The normal colonic epithelium of *Apc*^+/+^ and tumor samples from *Apc*^*Min*/+^ were included for additional quantitative comparisons of transcript levels. Additional RNA was obtained using the protocol described above (Qiagen). Each sample was run in triplicate and technical error between replicates did not exceed 5%. Fold-change expression was determined by calculating 2^n^ for each sample, where n equals the difference in amplification cycle between the GAPDH reference and the test probe. The parameter deltaCT gives the number rounds of amplification needed to reach threshold compared to the number required for the control GAPDH probe.

## Supporting information

S1 Table Test of effect of the *Pde4b* genotype on the number of adenomas in the small intestine of *Apc*^*Min*/+^ mice over a series of backcross-intercross generations. Gross tumor numbers were measured in the small intestines of *Apc*^*Min*/+^ mice from the series of backcross-intercross generations, carrying the *Pde4b+/+*, *Pde4b+/-* and *Pde4b-/-* genotypes.
(PDF)

S2 Table Results of gene-set enrichment analysis for 75 genes associated with increased expression in colonic adenoma in all three genera. Shown are enriched GO terms at z-score exceeding 5.0 (p < 10^-6^), set size at least 10 genes, and containing at least 3 adenoma-associated genes. Below, column set.mean is the proportion of the GO category that are triply conserved adenoma-associated genes. Computed with *allez* [70].
(PDF)

S3 Table Results of gene-set enrichment analysis for 14 genes associated with decreased expression in colonic adenoma in all three genera. Shown are enriched GO terms at z-score exceeding 5.0 (p < 10^-6^), set size at least 10 genes, and containing at least 3 adenoma associated genes. Below, column set.mean is the proportion of the GO category that are triply conserved adenoma-associated genes. Computed with *allez* [70].
(PDF)

S1 Fig Average (over mouse samples, per probe) of log2 expression level, for all probes (grey), probes identified as differentially expressed between tumor and normal samples in mouse (blue), and the subset of those that are consistently differentially expressed across in all genera (red). Boxplot width is proportional to the square root of the number of probes.
(PDF)

S2 Fig Average (over rat samples, per probe) of log2 expression level, for all probes (grey), probes identified as differentially expressed between tumor and normal samples in rat (blue), and the subset of those that are consistently differentially expressed across in all genera (red). Boxplot width is proportional to the square root of the number of probes.
(PDF)

S3 Fig Permutation distribution of the 3-way overlap of gene lists, compared to observed overlap (red), for various levels of filtering to remove low-expressing genes. Calculations are based upon 10,000 permutations in each case. Boxplots show median (black), interquartile range (grey), and whiskers extending to cover the central 95% of the permutation distribution.
(PDF)

S4 Fig Dot plots of the colon tumor count data summarized in Table 1. Medians are indicated by horizontal lines.
(PDF)

S5 Fig Real time PCR results for *Pde4b* transcripts of the normal colonic epithelium (NCE) whose level differ between *Apc*^*Min/+*^ mice bearing colonic adenomas (T+) and *Apc*^*Min/+*^ mice free of colonic tumors (T-). The cycle number (ΔCT), normalized to GAPDH, is shown on the Y axis. Each dot represents the average of triplicate values from an individual sample. Medians and quartiles for each tissue type are indicated. WT, wildtype.
(PDF)
