# Supplementary material for: The conserved protective cyclic AMP-phosphodiesterase function PDE4B is expressed in the adenoma and adjacent normal colonic epithelium of mammals and silenced in colorectal cancer
Source: PLoS Genet. 2018 Sep 6;14(9):e1007611. doi: 10.1371/journal.pgen.1007611 (PMC6143270; doi:10.1371/journal.pgen.1007611)
Supplement: S1 Table — Gross tumor numbers were measured in the small intestines of ApcMin/+ mice from the series of backcross-intercross generations, carrying the Pde4b+/+, Pde4b+/- and Pde4b-/- genotypes. (PDF) [file pgen.1007611.s001.pdf]

S1 Table. Test of effect of the *Pde4b* genotype on the number of adenomas in the small intestine of *Apc* $^{Min/+}$ mice over a series of backcross-intercross generations. Gross tumor numbers were measured in the small intestines of *Apc*$^{Min/+}$ mice from the series of backcross-intercross generations, carrying the *Pde4b*$^{+/+}$, *Pde4b*$^{+/-}$ and *Pde4b*$^{-/-}$ genotypes.

	Small intestinal tumor counts, mean ± SD (n of mice)		
Genotype	*Apc*$^{Min/+}$ *Pde4b*$^{+/+}$	*Apc*$^{Min/+}$ *Pde4b*$^{+/-}$	*Apc*$^{Min/+}$ *Pde4b*$^{-/-}$
F2	78.5 ± 32.4 (6)	81.8 ± 32.1 (13)	66.7 ± 24.0 (6)
N2F2	87.0 ± 34.1 (46)	93.5 ± 42.9 (86)	92.4 ± 41.0 (31)
N3F2	107.2 ± 28.6 (23)	117.5 ± 33.8 (27)	125.3 ± 36.2 (9)
N4F2	111.7 ± 43.7 (9)	125.9 ± 46.9 (15)	103.1 ± 35.7 (8)
