# Supplementary material for: The conserved protective cyclic AMP-phosphodiesterase function PDE4B is expressed in the adenoma and adjacent normal colonic epithelium of mammals and silenced in colorectal cancer
Source: PLoS Genet. 2018 Sep 6;14(9):e1007611. doi: 10.1371/journal.pgen.1007611 (PMC6143270; doi:10.1371/journal.pgen.1007611)
Supplement: S2 Table — Shown are enriched GO terms at z-score exceeding 5.0 (p < 10-6), set size at least 10 genes, and containing at least 3 adenoma-associated genes. Below, column set.mean is the proportion of the GO category that are triply conserved adenoma-associated genes. Computed with allez [70]. (PDF) [file pgen.1007611.s002.pdf]

S2 Table. Results of gene-set enrichment analysis for 75 genes associated with increased expression in colonic adenoma in all three genera. Shown are enriched GO terms at z-score exceeding 5.0 ($p < 1e-6$), set size at least 10 genes, and containing at least 3 adenoma-associated genes. Below, column set.mean is the proportion of the GO category that are triply conserved adenoma-associated genes. Computed with *allez* [70].

ID	Term	Ontology	set.mean	set.size	z.score
GO:2000052	positive regulation of non-canonical Wnt signaling pathway	BP	0.23077	13	11.807
GO:0001780	neutrophil homeostasis	BP	0.15789	19	9.673
GO:1902624	positive regulation of neutrophil migration	BP	0.15625	32	12.423
GO:0090023	positive regulation of neutrophil chemotaxis	BP	0.15385	26	11.018
GO:0071624	positive regulation of granulocyte chemotaxis	BP	0.14815	27	10.799
GO:2000050	regulation of non-canonical Wnt signaling pathway	BP	0.13636	22	8.945
GO:0090022	regulation of neutrophil chemotaxis	BP	0.13333	30	10.208
GO:1902622	regulation of neutrophil migration	BP	0.13158	38	11.335
GO:0003401	axis elongation	BP	0.10345	29	7.703
GO:0032743	positive regulation of interleukin-2 production	BP	0.1	30	7.561
GO:0071622	regulation of granulocyte chemotaxis	BP	0.08889	45	8.183
GO:0060412	ventricular septum morphogenesis	BP	0.08108	37	6.73
GO:0032729	positive regulation of interferon-gamma production	BP	0.08065	62	8.669
GO:0048146	positive regulation of fibroblast proliferation	BP	0.08065	62	8.669
GO:0002690	positive regulation of leukocyte chemotaxis	BP	0.07792	77	9.318
GO:1990266	neutrophil migration	BP	0.07229	83	8.931
GO:0030593	neutrophil chemotaxis	BP	0.06944	72	7.965
GO:0035567	non-canonical Wnt signaling pathway	BP	0.06667	45	6.021
GO:0002688	regulation of leukocyte chemotaxis	BP	0.06383	94	8.316
GO:0032663	regulation of interleukin-2 production	BP	0.06	50	5.664
GO:0002687	positive regulation of leukocyte migration	BP	0.05882	119	8.571
GO:0097530	granulocyte migration	BP	0.05825	103	7.885
GO:0032649	regulation of interferon-gamma production	BP	0.05556	90	6.996
GO:0071621	granulocyte chemotaxis	BP	0.05495	91	6.951
GO:0032623	interleukin-2 production	BP	0.05455	55	5.354
GO:0060986	endocrine hormone secretion	BP	0.05357	56	5.297
GO:0048145	regulation of fibroblast proliferation	BP	0.05319	94	6.818
GO:0042108	positive regulation of cytokine biosynthetic process	BP	0.05263	57	5.241
GO:0048144	fibroblast proliferation	BP	0.05263	95	6.775
GO:0032609	interferon-gamma production	BP	0.05051	99	6.61
GO:0050921	positive regulation of chemotaxis	BP	0.04959	121	7.165
GO:0045766	positive regulation of angiogenesis	BP	0.04918	122	7.13
GO:0060021	palate development	BP	0.04706	85	5.66
GO:0097529	myeloid leukocyte migration	BP	0.04698	149	7.495
GO:0002685	regulation of leukocyte migration	BP	0.0443	158	7.231
GO:0046660	female sex differentiation	BP	0.04386	114	6.065
GO:1904018	positive regulation of vasculature development	BP	0.04348	138	6.612
GO:0030595	leukocyte chemotaxis	BP	0.04217	166	7.013
GO:0005179	hormone activity	MF	0.04167	96	5.25

GO:0090575	RNA polymerase II transcription factor complex	CC	0.03883	103	5.021
GO:0046661	male sex differentiation	BP	0.03788	132	5.53
GO:0050920	regulation of chemotaxis	BP	0.03681	163	5.953
GO:0005125	cytokine activity	MF	0.03509	171	5.771
GO:0008406	gonad development	BP	0.03243	185	5.479
GO:0060326	cell chemotaxis	BP	0.03211	218	5.884
GO:0045137	development of primary sexual characteristics	BP	0.03209	187	5.439
GO:0002237	response to molecule of bacterial origin	BP	0.0307	228	5.709
GO:0007548	sex differentiation	BP	0.03017	232	5.642
GO:0050900	leukocyte migration	BP	0.02985	268	5.994
GO:0032103	positive regulation of response to external stimulus	BP	0.02857	245	5.435
GO:0032496	response to lipopolysaccharide	BP	0.02844	211	5.009
GO:0045765	regulation of angiogenesis	BP	0.02844	211	5.009
GO:0048608	reproductive structure development	BP	0.02488	402	5.91
GO:0061458	reproductive system development	BP	0.02463	406	5.868
GO:0030335	positive regulation of cell migration	BP	0.02445	409	5.836
GO:2000147	positive regulation of cell motility	BP	0.02375	421	5.714
GO:0051272	positive regulation of cellular component movement	BP	0.02315	432	5.605
GO:0040017	positive regulation of locomotion	BP	0.02227	449	5.445
GO:0044057	regulation of system process	BP	0.02193	456	5.381
GO:0002009	morphogenesis of an epithelium	BP	0.02053	487	5.113
