# Supplementary material for: The conserved protective cyclic AMP-phosphodiesterase function PDE4B is expressed in the adenoma and adjacent normal colonic epithelium of mammals and silenced in colorectal cancer
Source: PLoS Genet. 2018 Sep 6;14(9):e1007611. doi: 10.1371/journal.pgen.1007611 (PMC6143270; doi:10.1371/journal.pgen.1007611)
Supplement: S3 Table — Shown are enriched GO terms at z-score exceeding 5.0 (p < 10-6), set size at least 10 genes, and containing at least 3 adenoma associated genes. Below, column set.mean is the proportion of the GO category that are triply conserved adenoma-associated genes. Computed with allez [70]. (PDF) [file pgen.1007611.s003.pdf]

S3 Table. Results of gene-set enrichment analysis for 14 genes associated with decreased expression in colonic adenoma in all three genera. Shown are enriched GO terms at z-score exceeding 5.0 (p < 1e-6), set size at least 10 genes, and containing at least 3 adenoma-associated genes. Below, column set.mean is the proportion of the GO category that are triply conserved adenoma-associated genes. Computed with *allez* [70].

ID	Term	Ontology	set.mean	set.size	z.score
GO:0007605	sensory perception of sound	BP	0.02256	133	8.395
GO:0050954	sensory perception of mechanical stimulus	BP	0.01911	157	7.675
GO:0007623	circadian rhythm	BP	0.01676	179	7.144
GO:0015850	organic hydroxy compound transport	BP	0.01538	195	6.813
GO:0022804	active transmembrane transporter activity	MF	0.01242	322	6.994
GO:0016324	apical plasma membrane	CC	0.01103	272	5.642
GO:0048511	rhythmic process	BP	0.01031	291	5.425
