# Supplementary material for: The conserved protective cyclic AMP-phosphodiesterase function PDE4B is expressed in the adenoma and adjacent normal colonic epithelium of mammals and silenced in colorectal cancer
Source: PLoS Genet. 2018 Sep 6;14(9):e1007611. doi: 10.1371/journal.pgen.1007611 (PMC6143270; doi:10.1371/journal.pgen.1007611)
Supplement: S2 Fig — Boxplot width is proportional to the square root of the number of probes. (PDF) [file pgen.1007611.s005.pdf]

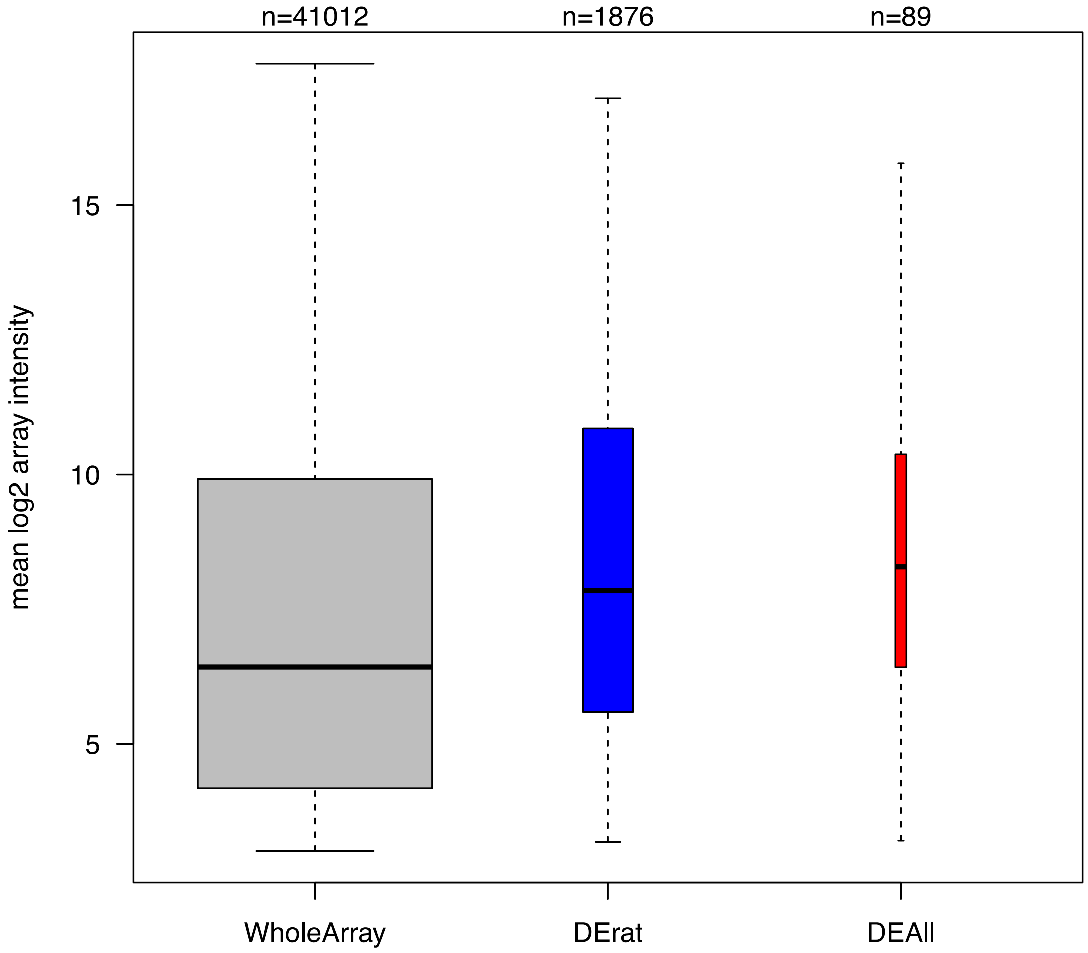

Figure 2. Average (over samples, per probe) of log2 expression level, for all probes (grey), probes identified as differentially expressed between tumor and normal samples in rat (blue), and the subset of those that are consistently differentially expressed across in all species (red). Boxplot width is proportional to the square root of the number of probes.
