# Supplementary figures and images for: The conserved protective cyclic AMP-phosphodiesterase function PDE4B is expressed in the adenoma and adjacent normal colonic epithelium of mammals and silenced in colorectal cancer
Source: PLoS Genet. 2018 Sep 6;14(9):e1007611. doi: 10.1371/journal.pgen.1007611 (PMC6143270; doi:10.1371/journal.pgen.1007611)

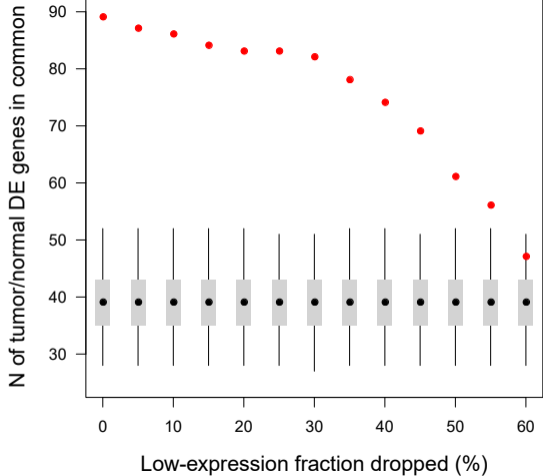

Supplement: S3 Fig — Calculations are based upon 10,000 permutations in each case. Boxplots show median (black), interquartile range (grey), and whiskers extending to cover the central 95% of the permutation distribution. (PDF) [file pgen.1007611.s006.pdf]
