# Supplementary material for: The conserved protective cyclic AMP-phosphodiesterase function PDE4B is expressed in the adenoma and adjacent normal colonic epithelium of mammals and silenced in colorectal cancer
Source: PLoS Genet. 2018 Sep 6;14(9):e1007611. doi: 10.1371/journal.pgen.1007611 (PMC6143270; doi:10.1371/journal.pgen.1007611)
Supplement: S4 Fig — Medians are indicated by horizontal lines. (PDF) [file pgen.1007611.s007.pdf]

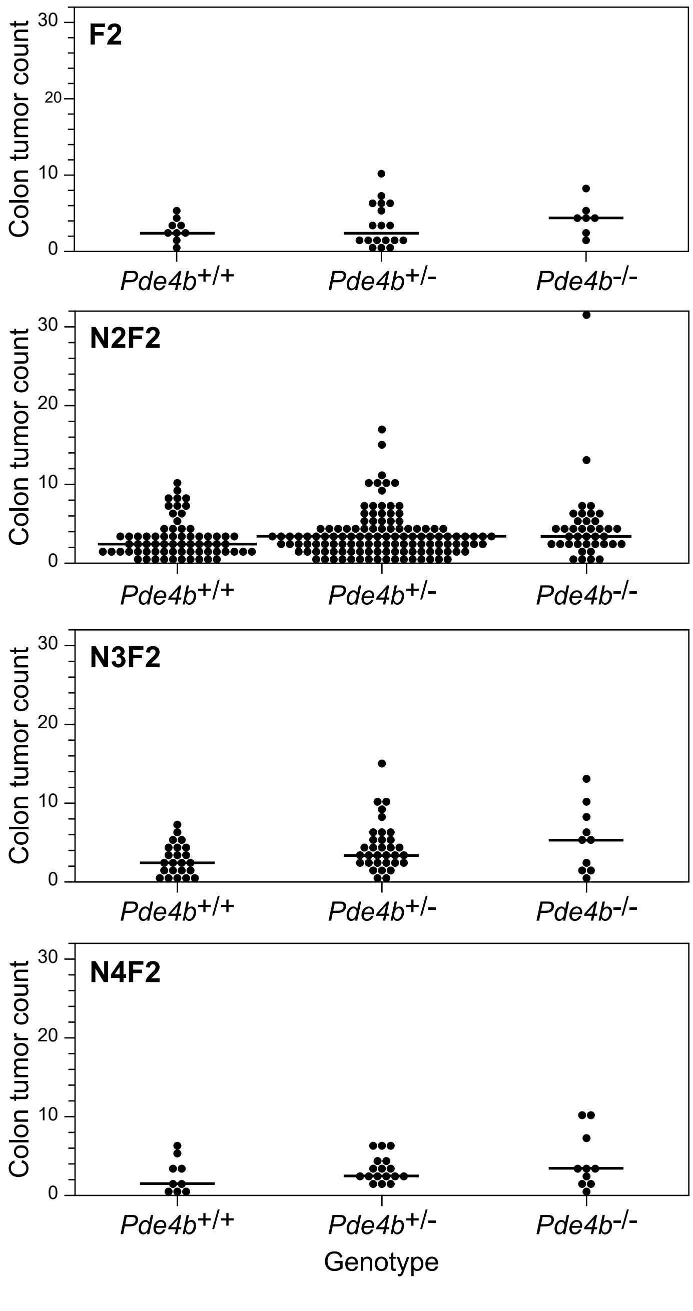

S4 Figure. Dot plots of the colon tumor count data summarized in Table 1. Medians are indicated by horizontal lines.
