# Supplementary material for: The conserved protective cyclic AMP-phosphodiesterase function PDE4B is expressed in the adenoma and adjacent normal colonic epithelium of mammals and silenced in colorectal cancer
Source: PLoS Genet. 2018 Sep 6;14(9):e1007611. doi: 10.1371/journal.pgen.1007611 (PMC6143270; doi:10.1371/journal.pgen.1007611)
Supplement: S5 Fig — The cycle number (ΔCT), normalized to GAPDH, is shown on the Y axis. Each dot represents the average of triplicate values from an individual sample. Medians and quartiles for each tissue type are indicated. WT, wildtype. (PDF) [file pgen.1007611.s008.pdf]

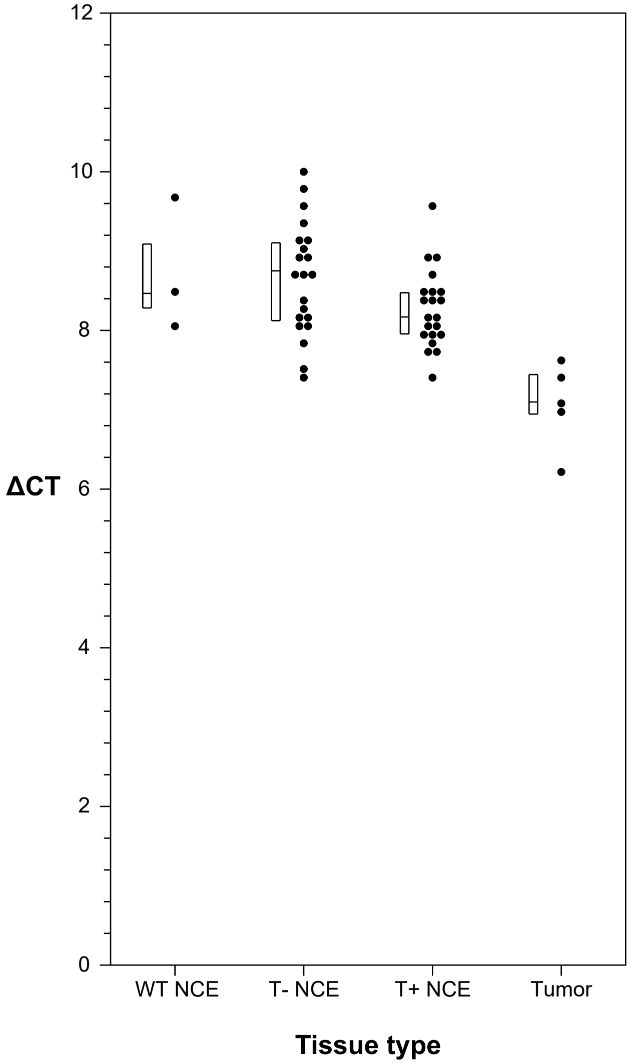

S5 Figure. Real time PCR results for transcripts of the normal colonic epithelium (NCE) whose level differ between *Apc*$^{Min/+}$ mice bearing colonic adenomas (T+) and *Apc*$^{Min/+}$ mice free of colonic tumors (T-). The cycle number (ΔCT), normalized to GAPDH, is shown on the Y axis. Each dot represents the average of triplicate values from an individual sample. Medians and quartiles for each tissue type are indicated. WT, wildtype.
